# Self-Care in Pediatric Patients with Chronic Conditions: A Systematic Review of Theoretical Models

**DOI:** 10.3390/ijerph18073513

**Published:** 2021-03-28

**Authors:** Immacolata Dall’Oglio, Giulia Gasperini, Claudia Carlin, Valentina Biagioli, Orsola Gawronski, Giuseppina Spitaletta, Teresa Grimaldi Capitello, Michele Salata, Valentina Vanzi, Gennaro Rocco, Emanuela Tiozzo, Ercole Vellone, Massimiliano Raponi

**Affiliations:** 1 Bambino Gesù Children’s Hospital, IRCCS, Piazza Sant’Onofrio 4, 00165 Rome, Italy; giulia.gasperini@uniroma1.it (G.G.); claudia.carlin@opbg.net (C.C.); valentina.biagioli@opbg.net (V.B.); orsola.gawronski@opbg.net (O.G.); giuseppina.spitaletta@opbg.net (G.S.); teresa.grimaldi@opbg.net (T.G.C.); michele.salata@opbg.net (M.S.); valentina.vanzi@opbg.net (V.V.); emanuela.tiozzo@opbg.net (E.T.); massimiliano.raponi@opbg.net (M.R.); 2 Department of Biomedicine and Prevention, University of Rome Tor Vergata, 00133 Rome, Italy; ercole.vellone@uniroma2.it; 3 Centre of Excellence for Nursing Scholarship-Nursing Professional Order of Rome, Viale Giulio Cesare, 78, 00192 Rome, Italy; genna.rocco@gmail.com

**Keywords:** self-care, model, chronic diseases, pediatric, young adults

## Abstract

Background: To improve outcomes in children and young adults (CYAs) with chronic conditions, it is important to promote self-care through education and support. Aims: (1) to retrieve the literature describing theories or conceptual models of self-care in CYAs with chronic conditions and (2) to develop a comprehensive framework. Methods: A systematic literature search was conducted on nine databases, according to the Preferred Reporting Items for Systematic Reviews and Meta-Analyses (PRISMA) guidelines. All peer-reviewed papers describing a theory or a conceptual model of self-care in CYAs (0–24 years) with chronic conditions were included. Results: Of 2674 records, 17 met the inclusion criteria. Six papers included a theory or a model of self-care, self-management, or a similar concept. Six papers developed or revised pre-existing models or theories, while five papers did not directly focus on a specific model or a theory. Patients were CYAs, mainly with type 1 diabetes mellitus and asthma. Some relevant findings about self-care in CYAs with neurocognitive impairment and in those living with cancer may have been missed. Conclusions: By aggregating the key elements of the 13 self-care conceptual models identified in the review, we developed a new overarching model emphasizing the shift of self-care agency from family to patients as main actors of their self-management process. The model describes influencing factors, self-care behaviors, and outcomes; the more patients engaged in self-care behaviors, the more the outcomes were favorable.

## 1. Introduction

A long-term chronic condition, often associated with medical complexity [1], “requires ongoing management over a period of years or decades” [2], with biological, psychological, or cognitive foundations lasting for at least one year and impacting wellbeing [3]. The most common pediatric chronic conditions include asthma, cystic fibrosis, type 1 diabetes mellitus, and chronic lung disease [4]. The number of children and young adults (CYA)—which we consider aged between 0–24 [5,6]—living with a chronic condition is growing thanks to higher survival rates [7]. In the United States, about 25% of the pediatric population is affected by a chronic condition, and 5% are affected by multiple chronic conditions [8]. In 2016, 16% of the European population aged between 16–29 years had a long-standing health problem [9]. In particular, the broadness of epidemiology and the variety of clinical conditions in CYAs are factors that have to be taken into account to ensure their best possible physical, emotional, and social development [10,11].

Chronic conditions may have a negative impact on children and their families. CYAs go through physiological developmental stages, which could influence their ability to deal with their condition and vice versa. Younger children fully depend on their family members, but as they grow up, they prefer to take care of themselves as they grow through puberty and develop their personal identity. CYAs with chronic conditions may develop psychological and behavioral problems, including low self-esteem, anxiousness, depression, anxiety [12,13], or other problems, which may result directly from the disease or indirectly from the awareness and the experience of illness [14]. In addition, somatic problems, social abandonment, aggression, and rage may lead to mental health problems or behavioral disorders [13]. Another negative outcome is related to school and academic performance, such as persistent absence and poorer performance, which might undermine self-esteem [13,15]. However, during the personal development process, peer groups play an important role in improving CYAs’ quality of life and their ability to cope with their chronic condition.

The chronic condition and the perception of behavioral problems of a child are not solely related to the child but affect the lifestyle of all the family members, including parents or siblings who are likely to experience stress [16,17]. Caring for a child with a chronic condition can also undermine parents’ job status, such as the number of work hours and maternal employment [18,19]. Having a child with a chronic condition can also decrease parents’ leisure time [18,20]. Furthermore, parents could encounter more difficulties if they lack moments of relief, adequate coping skills, and enough social and community support [13].

Several additional family factors could worsen the general health in children with disabilities. Some of these are described in the literature as caregiver burden, limited interactions with extended family and friends, or economic problems and family conflict [13,21]. In this type of family context, CYAs can experience negative health outcomes including stress and poor adjustment, poorer coping skills, and higher hospitalization rates [13,21]. To support these parents, parenting support programs based on preventive psychology could reduce emotional fragility in parents of CYAs with chronic conditions [22,23].

Scholars suggest that, to improve health outcomes in children with chronic conditions, it is crucial to promote their self-care or self-management through education and support both for patients and their families [11,24,25]. Since 1985, Orem, in her model, uses self- or dependent-care deficit as a useful basis for the promotion of self-care in the chronically ill pediatric population, where the focus is on the caring relationship [26]. Self-management is described as “the interaction of health behaviors and related processes that patients and families engage in to care for a chronic condition” [27]. This concept is also referred to as self-care, defined by the World Health Organization as “the ability of individuals, families and communities to promote, maintain health, prevent disease and to cope with illness and disability with or without the support of a healthcare provider” [28].

To our knowledge, although sound theories of self-care have been developed for adult patients with chronic conditions and their caregivers [29,30,31,32], no comprehensive review has evaluated which theory or conceptual model fits best for CYAs living with chronic conditions. In addition, there are no other systematic reviews on this topic.

Given the particular characteristics of this population and the need to adopt a family perspective, it seems appropriate to consider a conceptual model/theory drawn directly from a CYA context rather than adapting one from an adult context. A comprehensive model could guide multi-professional interventions aimed at promoting self-care, for example, by improving education for CYAs and/or facilitating the coordination of integrated care for these patients.

Therefore, the aims of this study were: (1) to retrieve the literature describing theories or conceptual models of self-care involving CYAs living with chronic conditions; and (2) to develop a comprehensive framework specific for CYAs with chronic conditions, taking into account the key elements of previous theories or conceptual models.

## 2. Methods

This is a systematic literature review that includes studies regarding theories or conceptual models of self-care in CYAs living with chronic conditions.

### 2.1. Search Strategy

Due to the specific topic of this review, only the PICOS (Population, Intervention, Comparison, Outcomes and Study design) keywords concerning population and intervention were used to identify as many relevant articles as possible [33]. Before starting the review, a protocol was developed [34,35]. The following databases were searched from inception to July 2019, PubMed, Scopus, Cochrane Library, Cumulative Index to Nursing and Allied Health Literature (CINAHL), EMBASE, Web of Science, Joanna Briggs Institute (JBI), PsycINFO, and PsycARTICLES. The search was performed by two researchers independently (G.G and C.C).

The main keywords were self-care, self-monitoring, self-management, self-maintenance, chronic conditions, pediatric (0–18 years), and young adults (19–24 years). Boolean operators and truncations were used with different combinations across the nine databases. No limits were selected. Additional studies were identified through other sources and by handsearching the reference lists of the included studies. The full search strategy is shown in Supplementary File S1.

### 2.2. Eligibility Criteria

The review included all types of peer-reviewed papers with no limits of time or language. To be eligible, papers had to include the description of a theory or a conceptual model of self-care in the context of CYAs (0–24 years, according to PubMed limits and the World Health Organization and United Nations’ definition of “youth”) [5,6] with chronic conditions. Papers were excluded if the theory was only briefly mentioned and not sufficiently described to gain a deep understanding of the relationships among the key factors. However, the seminal studies of specific self-care theories cited in those papers were searched manually and included in the review process. Grey literature and studies involving adults older than 24 years were excluded. Studies involving only children with neurocognitive impairment were excluded considering the difficulties these children could experience with self-care behaviors. In addition, studies involving only children with cancer diagnosis were excluded because they may need to adopt different self-care strategies to help them cope with a life-threatening disease.

### 2.3. Study Selection

The Preferred Reporting Items for Systematic Reviews and Meta-Analyses (PRISMA) Guidelines were followed. [33] G.G and C.C identified the duplicate records and removed them before the screening process. They independently examined all the titles of the retrieved the records. When the titles were considered relevant, also the abstracts were read. Then, the full texts of relevant abstracts were read and critically reviewed. In case of disagreement between the two researchers, a third researcher was involved.

### 2.4. Data Extraction and Synthesis

Data from the included studies were examined and synthesized to analyze the models and the theories of self-care in CYAs living with chronic conditions. At the end of the data screening process, the following information was collected from each paper: author names, aim of the study, country where the study was conducted or the authors’ nationality if the study did not involve data collection, sample characteristics (age and chronic conditions), study design, and a theory or a conceptual model. Specific information about the theories and the conceptual models were recorded, such as the name of the theory or the conceptual model, the principles, the core components, the type of theory or conceptual model, the influencing factors, and the outcomes. If there were several versions of the same theory or conceptual model from the same authors, only the most recent one was taken into account. This choice was made to facilitate the understanding of the relevant theories/models identified through this review.

Data were then aggregated to generate an overarching model of self-care in CYAs living with chronic conditions, differentiating between influencing factors, self-care behaviors, and outcomes. The researchers attempted to identify similarities and differences across theories/models. A synthesis of at least two similar key elements was performed through the generation of a concept describing the main finding.

## 3. Results

### 3.1. Flow Diagram

**Figure 1:**
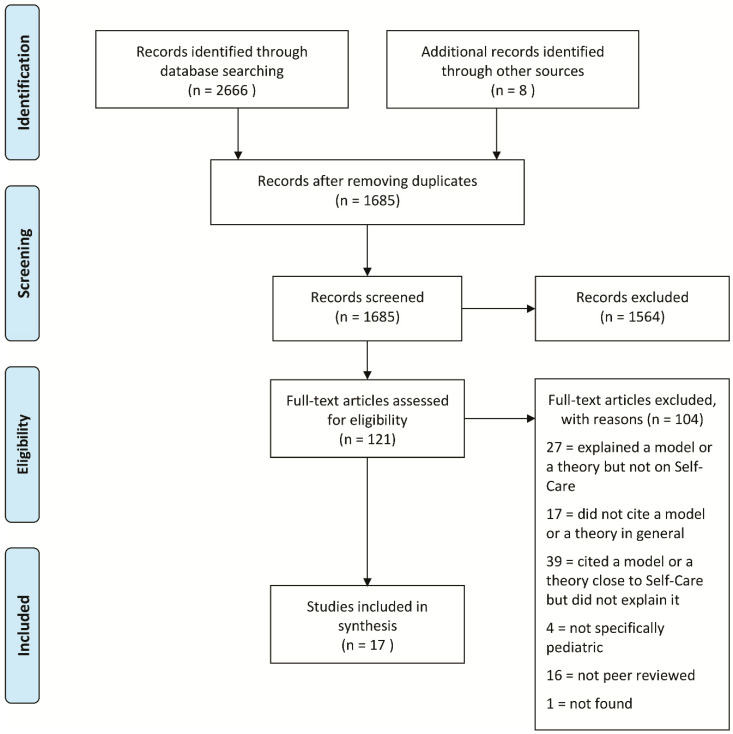
Preferred Reporting Items for Systematic Reviews and Meta-Analyses (PRISMA) flow diagram.

The PRISMA flow diagram of the entire process of study identification, screening, eligibility, and inclusion is shown in Figure 1. From the databases, 2666 records were retrieved (PubMed: *n* = 277; Scopus: *n* = 317; Cochrane: *n* = 280; CINAHL: *n* = 351; EMBASE: *n* = 565; Web of Science: *n* = 396 JBI: *n* = 77; PsycINFO: *n* = 359; and PsycARTICLES: *n* = 44) and 8 additional records were collected from other sources. A total of 1685 records were examined after duplicates were removed. Among these, 1564 were excluded after reading the titles and/or abstracts, and 121 full texts were examined.

### 3.2. Characteristics of the Included Studies

In the 17 included studies, various theories or conceptual models about self-care in CYAs living with chronic conditions were presented. The characteristics of the included studies are summarized in Table 1. Six (35.3%) papers had the aim of introducing a theory or a model of self-care or a similar concept [27,36,37,38,39,40]; six (35.3%) papers continued or revised pre-existing models or theories [41,42,43,44,45,46]; and five (29.4%) did not specifically have the aim of illustrating a model or a theory but included one anyway [47,48,49,50,51]. Most of the studies were conducted in the United States (*n* = 14; 82.3%) [27,36,37,39,40,41,42,43,45,46,48,49,50], two in Europe (*n* = 2; 11.8%) [38,47], and one (5.9%) described a sample from four continents [44]. The 17 included studies had a wide range of designs, including grounded theory (*n* = 3; 17.6%) [47,49,50], concept analysis (*n* = 1; 5.9%) [40], and systematic reviews (*n* = 3; 17.6%) [43,44,48].

The characteristics of the study samples including both age and chronic conditions were described in six (35.3%) papers [37,38,41,47,49,50], and the age range was between 1–18 years. With regard to the types of chronic conditions, in six (35.3%) papers, the sample was affected by one chronic condition, either type 1 diabetes mellitus [38,46,47] or asthma [37,49,51]. In four (23.6%) studies, the sample was affected by various chronic conditions [41,43,44,50], and in the remaining studies, the authors did not specify the chronic conditions of their participants. In two articles (11.8%), the sample included toddlers and pre-school children (1–15 years old) affected by asthma [37,49]. In three studies, the sample included adolescents (13–18 years old); in two studies (11.8%), they were affected by type 1 diabetes mellitus [28,37] and in one (5.9%) by type 1 diabetes mellitus and other chronic conditions [50]. Moreover, one study (5.9%) included a sample of schoolers and pre-adolescents (8–13 years old) with various chronic conditions, such as asthma, type 1 diabetes mellitus, or cystic fibrosis [41]. The other papers (*n* = 11; 64.6%) did not include any information about the age of their sample. Of the included papers, six (35%) reported data on the gender distribution of their sample. In most of these studies, gender distribution was even [40,41,47,49]; one study included mainly male patients [37] and another one mainly female participants and their mothers [50].

### 3.3. Findings about Theories or Conceptual Models

Most of the studies described conceptual models from qualitative studies, reviews, or adaptation from others. Only two quantitative studies tested the previous conceptual models in a group of patients [37,38].

In four studies (23.5%), “The Family Management Style Framework (FMSF)”, about how families and children manage their illness, was described [40,41,43,44]. In two papers (11.8%), the “Self- and Family Management Framework” was defined, illustrating both the self-management and the family management of the chronic conditions [39,42]. Three studies (17.6%) focused on “Self-Regulation”, described as a way of managing chronic illnesses [37,51] or linked it to the Self-Management Framework by Modi et al. (2012) [27,48]. Sonney and Insel (2016) [45] examined the Common Sense Model of Parent–Child Shared Regulation, which refers to illness and self-regulatory plans as shared processes. Williams-Raede et al. (2019) [50] presented the “Theory of Parent–Child Relational Illness Management”, describing how parents and children influence each other.

In two studies (11.8%), the authors described the adaptive process to type 1 diabetes mellitus through two different models, one that considered adaptation as the final goal in caring for oneself [46] and the other that considered it as a process that flows from difficulty to success in self-management [47]. Beacham and Deatrick (2013) [36] adapted a theory of self-care for adults with chronic illnesses to the pediatric context and included it in the Health Care Autonomy framework. Modi et al. (2012) [27] developed the “Pediatric Self-Management Framework”, which focused on the process of self-management, the elements that influence it, and its outcomes. Shaw and Oneal (2014) [49] presented the theory “Living on the edge of asthma”, which focuses on monitoring and managing symptoms and exacerbations. Kyngas (1999) [38] defined a theoretical model of compliance in young diabetics, which emphasizes what children do to maintain their health. The key elements of the previously described theories or conceptual models retrieved through this literature review are summarized in Table 2.

Of all the theories or conceptual models we retrieved through this systematic review, six papers (35.3%) involved “family members” or “family units” [36,41,44,46,47,49] and only two (11.8%) explicitly considered siblings and their relationship with CYAs with chronic conditions [27,48]. Moreover, Sonney and Insel (2016) [45] used the term “parent” referring to “an adult who is the primary caregiver of the child”. However, all the theories or the conceptual models considered the influence and the role of parents in the CYA self-care process. Two authors reported both the history of the child’s condition and the past experiences of the family described as elements that influence self-care [49,50].

The psychological factors that could influence self-care were mentioned in all the described theories. Besides, spiritual/religious aspects and contexts were explicitly mentioned in only two papers (11.8%) [27,42]. Most theories or conceptual models focused on the social context, with the exception of two studies [45,50]. For example, the school as a factor that influences the self-care process was reported in eight theories or conceptual models [27,36,37,41,42,47,48,49], and peer groups or friends were often taken into consideration [27,36,38,41,46,48,49]. The authors of some studies described family lifestyles as important influencing factors [27,36,37,41,42,44,47,49]. In addition, family culture, including traditions, beliefs and ethnicity were described in few studies [27,37,42,46]. Finally, among the explored theories, the importance of competence in navigating the healthcare system was mentioned in three studies [37,42,47].

### 3.4. Development of a Model of Self-Care in CYAs with Chronic Conditions

**Figure 2:**
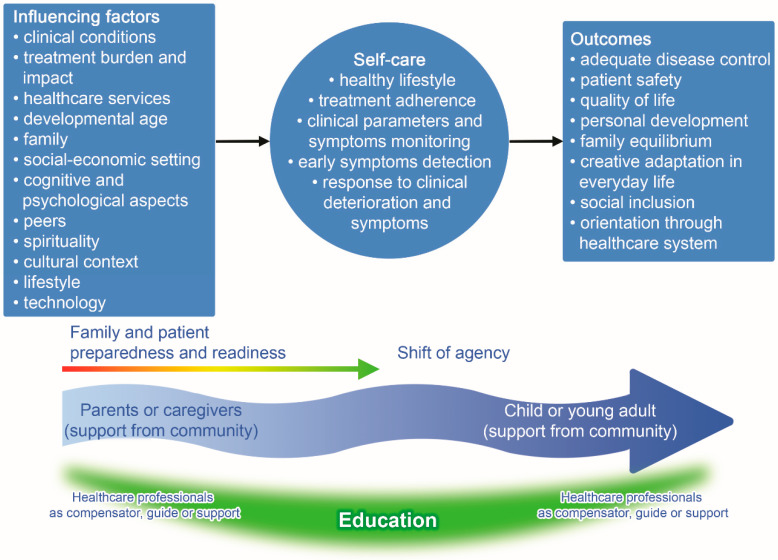
The comprehensive model of self-care in CYA with chronic conditions.

We considered the key elements of each of the 13 conceptual models and developed an overarching model considering the shift of agency from the family members to the CYAs, who become the main managers of their own self-care process (Figure 2). This shift is described as a dynamic process associated with developmental age, cognitive capabilities/readiness, and family preparedness to hand over this agency to their CYA. The self-care antecedents are factors that influence the level of CYA engagement and include: (1) condition-related factors, such as disease severity and treatment complexity, time since illness onset, and occurrence of acute events; (2) contextual social factors, such as socio-economic status and environmental issues; (3) contextual family factors, such as the presence of another family member with a chronic condition and the educational level of family members; (4) psycho-spiritual aspects, such as religious beliefs/sense of control and beliefs about illness; and (5) general context factors related to culture, lifestyle, and the characteristics of healthcare services.

Self-care behaviors include: (1) self-care daily activities to achieve healthy lifestyles, adhere to the prescribed treatment, and keep one’s own health status under control; (2) constant monitoring of clinical parameters, symptoms, and evaluating risk status; and (3) ability to safely manage acute events or emergency situations.

The more a patient engaged in self-care behaviors, adopting effective behaviors, the more the results were likely to be favorable: (1) improved patient safety and disease control; (2) quality of life, considering both personal and family perspectives; (3) age-related personal development in all its dimensions, such as age-related development and cognitive development; (4) creative adaptation in everyday life, ability to improve social inclusion, and (5) adequate navigation through the healthcare system.

## 4. Discussion

The purpose of this review was to provide an overview of available theories and conceptual models describing self-care in children and young adults living with a chronic condition. This review enabled confirmation of the existence of 13 self-care models or theories, such as the development of health care autonomy, a conceptual model about the development of health care autonomy in children living with chronic conditions [36], Adapted Family Management Style Framework, the family management style framework, and self and family management framework revised, conceptual models about how families and children deal with the management of children’s chronic health conditions. [41,42,44]

This review included three conceptual frameworks for adolescents with type 1 diabetes mellitus: type 1 diabetes adaptation and self-management model, Childhood Adaptation Model to Diabetes Mellitus, and the theoretical model of compliance in young diabetics [38,46,47].

Furthermore, we found a grounded theory for children and adolescents (aged 2–15 years) with asthma and their families, Living on the Edge of Asthma [49], a conceptual model for pediatric patients with asthma aged 1–12 years, and a model of self-regulation for control of chronic disease [37].

A conceptual model about self-regulation in adolescents living with chronic conditions was found: adolescent self-regulation as a foundation for chronic illness self-management [48].

A comprehensive model of self-management for pediatric patients was found, the Pediatric Self-management Model [27], and a theory based on “Common sense model of self-regulation of health and illness” for pediatric asthma, the common sense model of parent–child shared regulation [45]. Finally, we found a theory of parent–child relational illness, and the theory of parent–child relational illness management [50]. We identified several frameworks that take into account the peculiarity of this population, the outcomes for the entire family, and the factors affecting the self-care process. The chronic conditions that were most frequently reported in the studies were asthma and type 1 diabetes mellitus. One reason could be the high prevalence of these chronic health conditions in the young population [11,57,58,59]. Another explanation could be the unmet needs to address and prevent the onset of acute exacerbations of these conditions [60], which sometimes constitute a main element of a theory or a conceptual model [49]. Many theories and conceptual models have the purpose of helping CYAs living with chronic conditions to achieve the best possible level of development, quality of life, and social integration, despite their chronic condition, across different contexts of life. For this reason, almost all of the theories take in account other contexts of life considering also peer groups, especially with adolescents [27,38,46,49].

This review pulled together the main factors related to self-care, which is often described as an ever-changing process according to external and internal influencing factors and outcomes. It is worth noting that almost all the studies were conducted in middle and high-income countries, such as the USA and Europe. Only one review study included a sample from Asia and South America in addition to Europe and Australia [44], and no study was retrieved from Africa. Since our search strategy did not have any limits on the language of the papers, we may conclude that self-care is typically studied in middle and high-income countries.

Since the study designs were mainly qualitative, the sample sizes were small, whereas the two quantitative studies had large samples. The major limitation of the included articles was that sometimes essential information about the sample characteristics was not reported, such as gender. In other studies, the sample was not equally distributed between males and females. Another important piece of information that was often missing was the length of time since diagnosis, even though this is a crucial factor in the process of becoming an expert in self-care [61]. Moreover, many studies were conducted in the 1990s [38,40,51], therefore, they may not fully reflect the evolution of healthcare and today’s cultural and social context.

The theories and the conceptual models that emerged from the included studies present a wide panorama of self-care in CYA patients living with chronic conditions and present different perspectives. Nevertheless, some weaknesses were found in the theories and the conceptual models. For example, only some of these explicitly mentioned siblings and family members other than parents [27,36,41,44,45,46,47,48,49]. Instead, persons other than family members or those present in the everyday social context were very important in facilitating the self-care process in CYAs living with chronic conditions [62]. Moreover, proactive family support was beneficial also for its members by playing an active role in the self-care process and not simply being the spectators of the lives of their loved ones [63]. Similarly, support coming from everyday social life made up of friends, peers, sport mates, and coaches could significantly improve the level of self-care and contribute to the process of inclusion into peer-groups [64,65]. This was pivotal to facilitate CYAs’ ability to cope with their chronic condition and improve their quality of life [66,67]. Finally, self-confidence, related to the self-care process, could improve the safety of CYAs with chronic conditions in every context, a process that involves both family members and other significant persons [68], as already described in adult patients [69]. In particular, the health system could support the families in carrying out the activities of daily living [11].

We also noticed that spiritual influence did not seem to be appropriately considered in the included studies. However, personal emotion and consciousness of one’s own spiritual interiority could represent a very important element in fostering self-care [70,71].

In addition, family needs seemed to be emphasized without taking into account the CYAs’ ability or potential to successfully take care of themselves assuming a “non-independent” approach. In fact, Coyne et al. (2013) [72] described that other scholars tended to consider the CYA as a unit within the family without uniquely describing the role of the CYA in the self-care process. Considering the evolution in the treatment of chronic conditions and of the social context, it would be necessary to develop a specific and comprehensive theory to capture in its entirety the phenomenon of self-care behavior in CYAs affected by chronic conditions.

The proposed model underlines the shift of agency from the family to CYAs, highlighting the educational role of healthcare professionals. This is a crucial element of the self-care agency transfer between three key actors: healthcare professionals, parents, and CYAs. The end stage of this process is that CYAs become autonomous and responsible for their self-care to improve the outcomes related to their chronic condition [52]. Every new educational intervention aimed at improving self-care behaviors needs to build on what learners already know, know how to do, and feel. Moreover, a welcoming community could be of great support in promoting emotional well-being and self-care both for parents and CYAs.

The present review demonstrates the constant attention scholars have when they address the problem of self-care in CYAs affected by a chronic condition. The various theories that have been described focus on various clinical, family, and social aspects of self-care. The proposed model of self-care is characterized by the fact that it combines all these aspects in the light of the new treatments available for these patients with the purpose to improve their quality of life and that of their families.

This model, directed at empowering patients with chronic condition and their family members, can be used to help healthcare and social professionals provide more appropriate and targeted educational interventions. In addition, it helps to gain a better understanding of the key role played by each life setting, starting from the school, in order to foster the normal growth and development of these children, despite their illness.

Finally, the awareness of this self-care model by peers, also in terms of formal associations (family associations), facilitates the collective action of support and advocacy and consequently a cultural change.

The model proposed in this study highlights how self-care is also the result of the family’s contribution to self-care.

This makes the models of self-care particularly complex, because they need to combine many different aspects that involve both CYAs and their families.

Therefore, the support and the assessment of self-care need to consider the relationship between the chronically ill children and their families [73,74,75].

### Limitations of the Literature Review

This systematic literature review has a few limitations. In the search process, the grey literature was not considered, thus relevant findings may have been missed. In addition, we decided not to include studies focusing on self-care in CYAs with neurocognitive impairment and in those living with cancer. Although we believe these patients deserve specific considerations, we may have missed some important features of the self-care process. Another limitation is that the evaluation of the methodological quality of each included article was not undertaken; this was mainly due to the multiple study designs. In the selection process, we included also studies with a small sample size and those with an incomplete description of the sample characteristics. Finally, the components identified in each theory were extremely diverse, therefore, it was difficult to determine the weight to ascribe to each component by following a rigorous approach, in line with other authors [76]. Further studies are recommended to confirm this comprehensive model of self-care in CYAs with chronic conditions.

## 5. Conclusions

This review provides an overview of the theories and the conceptual models that describe self-care in CYAs living with a chronic condition, especially those with asthma or type 1 diabetes mellitus. The key elements of the self-care process described in the included papers were aggregated into a new comprehensive model emphasizing the shift of the self-care agency from the family members to the CYAs, who become the main actors of their own self-care process. The model describes influencing factors, self-care behaviors, and outcomes; the more the patients engaged in self-care behaviors, the more the outcomes were favorable. This comprehensive model offers a global view of the world surrounding CYAs with chronic conditions. Therefore, it is necessary to ensure that CYAs receive self-care oriented support by a multi-professional team during their developmental ages. In fact, CYAs face great personal challenges and changes associated with their chronic conditions, which could affect their personal, family, and social life. The comprehensive model proposed in this study could enhance the awareness and the understanding of the CYA self-care process in healthcare professionals who are in the frontline to compensate, guide, and support the self-care process in CYAs and their families. In addition, this new model could facilitate the development of self-care interventions to promote self-care in CYAs and empower patients and their families to successfully manage self-care. Further studies are recommended to expand, contextualize, and validate this model and explore self-care processes in low-income countries.

## Figures and Tables

**Table 1:** Characteristics of the included studies.

Authors	Aim of the Study	Country	Study Design	Population *n*, Age, Gender	Population Chronic Condition	Results Theory or Conceptual Model
Beacham L.B., Deatrick J.A. (2013) [36]	To describe a developmental and family-based model of health care autonomy that incorporates self-care and family management. To apply the model to two case studies.	U.S.A.	NR	NR	NR	Development of Health Care Autonomy
Beacham L.B., Deatrick J.A. (2019) [41]	To adapt the Family Management Style Framework (FMSF) including the perspectives of children with chronic health conditions.	U.S.A. [41]	NR	Patients: *n* = 32 age = 8–13 gender: 56% male; 44% female [52]	Asthma, diabetes, cystic fibrosis, hemophilia, hereditary spherocytosis, phenylketonuria, sickle cell disease, eosinophilic gastrointestinal disease, chronic sinusitis [52].	Child Adapted FMSF
Chilton R, Pires-Yfantouda R. (2015) [47]	To describe how adolescents adapt their self-management requirements from diagnosis to self-management.	U.K.	Social Constructivist Grounded Theory Study.	Patients: *n* = 13 age = 13–17 gender: 54% male; 46% female	Type 1 diabetes mellitus.	Type 1 diabetes adaptation and self-management model
Clark N.M., Starr-Schneidkraut N.J. (1994) [51]	To describe asthma-management outcomes resulting from interventions for patients.	U.S.A.	NR	NR	Asthma.	Self-regulation: a model of patient management of asthma
Clark N.M., Gong M., Kaciroti N. (2014) [37]	To update a model for prevention and management of asthma focusing on the concept of self-regulatory processes.	U.S.A.	Observational Quantitative Study	Patients: *n* = 637 age = 1–12 gender: 70% male; 30% female Parents: *n* = 637 gender: NR	Asthma.	A Model of Self-Regulation for Control of Chronic Disease
Grey M., Knafl K., McCorkle R. (2006) [39]	To describe a framework focusing on the influencing factors and outcomes of self and family management.	U.S.A.	NR	NR	NR	Self and Family Management Framework
Grey M., Schulman-Green D., Knafl K., Reynolds N.R. (2015) [42]	To update the Self and Family Management Framework with new empirical, synthetic, and theoretical work.	U.S.A.	NR	NR	NR	Self and Family Management Framework (Revised)
Knafl K., Deatrick J.A. (1990) [40]	To analyze the concept of family management styles (FMS) as it relates to families in which there is a chronically ill or disabled child.	U.S.A.	Sartori’s approach for Concept Analysis [53].	Patients: *n* = 2 age = 11–12 gender: 50% male; 50% female Parents: *n* = 4 gender: 50% male; 50% female Siblings: *n* = 2 gender: 50% male; 50% female	NR	Family Management Style
Knafl K.A., Deatrick J.A. (2003) [43]	To describe current efforts to expand the FMSF.	U.S.A.	Comprehensive Review.	NR	A variety of illness situations including cancer, diabetes, asthma, and ventilator dependence.	FMSF (Revised)
Knafl K.A., Deatrick J.A., Havill N. (2012) [44]	To update the FMSF by further elaborating the eight dimensions.	Asia, Australia, Europe, or South America *	Review of research reports.	NR	The conditions included both chronic illnesses (e.g., asthma, Type 1 diabetes) and disabilities (e.g., cerebral palsy, spina bifida).	FMSF (Revised)
Kyngas H. (1999) [38]	To describe a theoretical model of compliance in young diabetics.	Finland	Grounded theory. Scale validation study. Cross-sectional study to validate the model. Qualitative data were used to expand the model.	Patients for study phases: *n* = 51; age = 13–17 *n* = 91 age = 12–17 *n* = 346 age = 13–17 gender: NR	Type 1 diabetes mellitus.	The theoretical model of compliance in young diabetics
Lansing A.H., Berg C.A. (2014) [48]	To describe the role of self-regulation as a foundation for individual and interpersonal sources of risk and resilience for chronic illness self-management in adolescents.	U.S.A.	Literature review	NR	NR	Adolescent Self-Regulation as a Foundation for Chronic Illness Self-Management.
Modi A.C., Pai A.L., Hommel K.A., Hood K.K., Cortina S. et al. (2012) [27]	To propose a comprehensive pediatric model of self-management.	U.S.A.	NR	NR	NR	Pediatric Self-management Model.
Shaw M.R. et Oneall G. (2014) [49]	To develop a grounded theory to guide interventions to reduce unnecessary hospitalizations and emergency department visits.	U.S.A.	Corbin and Strauss’s approach for Grounded Theory [54]	Patients: *n* = 10 age = 2–15 gender: 50% male; 50% female Parents: *n* = 13 gender: NR	Asthma.	“Living on the edge of asthma” theory.
Sonney J.T., Insel K.C. (2016) [45]	To update the Common Sense Model incorporating parent–child shared regulation of pediatric asthma.	U.S.A.	Fawcett’s framework for analysis and evaluation of nursing theories [55]	NR	NR	Common Sense Model of Parent–Child Shared Regulation (adapted from Leventhal et al. 2003) [56].
Whittemore R., Jaser S., Guo J., Grey M. (2010) [46]	To update the Childhood Adaptation Model to Chronic Illness for type 1 diabetes and to discuss research and clinical implications of the updated model.	U.S.A.	NR	NR	Type 1 diabetes mellitus.	Childhood Adaptation Model to Chronic Illness: Diabetes Mellitus (Revised)
Williams-Reade J.M., Tapanes D., Distelberg B.J., Montgomery S. (2019) [50]	To explore the unique challenges that adolescent patients and parents experience in relation to illness management.	U.S.A.	Qualitative Study, Grounded Theory Analysis	Patients: *n* = 16 age = 13–18 gender: 6% male; 94% female Parents: *n* = 16 gender: 25% male; 75% female	Type 1 diabetes mellitus; chronic pain; conversion disorder; genetic neurological disorder; migraines; genetic blood disorder; dwarfism.	Theory of Parent–Child Relational Illness Management

NR = not reported. * These countries are referred to the samples included in the studies analyzed in the review.

**Table 2:** Theories or conceptual models of self-care in children and young adults (CYAs) emerged from included studies.

Theory or Conceptual Model	Principles	Key Elements	Type of Theory or Conceptual Model	Influencing Factors	Outcomes	Details
Development of health care autonomy [36]		Condition management: Family management [44] Self-care (self-maintenance, self-monitoring, self-management) [20]	Conceptual model about development of health care autonomy in children living with chronic conditions.	Child readiness Parent readiness Interaction between parent and child	Health care autonomy and self-care Child health and well-being	
Adapted Family Management Style Framework [41]		Family management pattern: definition of situation (child identity, view of the condition, family mutuality) management behaviors (family philosophy, management approach) perceived consequences (family focus, future expectations)	Conceptual model about how families and children deal with condition management of children’s chronic health conditions	Contextual influences: Social support Care providers and systems Resources	Individual child outcomes Caregiver/parent outcomes Family unit outcomes	The authors adapted the Family management Style Framework [34] to include children’s views about themselves and their families
Family management style framework [44]	The perceived consequences shape management behaviors and affect the subsequent definition of the situation. Family: person with chronic condition and family members	Family management style: definition of situation (child identity, illness view, management mindset, parental mutuality) management behaviors (parenting philosophy, management approach) perceived consequences (family focus, future expectations)	Conceptual model about how families deal with condition management of children’s chronic health conditions	Contextual influences: Social network Care providers and systems Resources	Individual functioning Family unit functioning	
Type 1 diabetes adaptation and self-management model [47]	Self-management is a complex adaptive process within the continuum from difficulties to success.	Difficulties with self-management. Process mechanism. Transitional phases. Successful self-management.	Conceptual framework for adolescents with type 1 diabetes mellitus aged 13–17 years.	Blood glucose monitoring, existing parental involvement, accommodating school, integrating diabetes around others and in the future.	Taking ownership Becoming independent Perceived difficulty Prioritizing diabetes Exposing diabetes to others Achieving success Being challenged Utilizing incentives Momentum	
Childhood Adaptation Model to Diabetes Mellitus [46]	Adaptation is the final goal of caring for themselves.	Individual and family characteristics. Psychological responses. Individual and family responses. Adaptation.	Conceptual model for children with type 1 diabetes mellitus (age, NR).	Age, sex, duration of diabetes, socioeconomic status, race/ethnicity, treatment modality, pubertal development, family environment	Metabolic control Quality of life	Over the years, they conducted a series of studies on the efficacy of a coping skills training program and updated the model to include current research.
Living on the Edge of Asthma [49]	There is no order between balancing, losing control, seeking control, and transforming. These categories exist all in a continuous process and are interlinked.	On the edge of asthma	Grounded Theory for children and adolescents (aged 2–15 years) with asthma and their families.	Balancing Losing control Seeking control Transforming		The theory attempts to explain the process of families whose child had an asthma attack and was hospitalized or accessed to an emergency department.
A model of self-regulation for control of chronic disease [37]	Observations, judgments and reactions are the fulcrum of the self-regulation process.	Internal and external factors. Self-regulation. Management strategies. Endpoints.	Conceptual model for pediatric patients with asthma aged 1–12 years.	Intrapersonal resources (knowledge, attitudes, feelings, beliefs) External resources (role models, technical advice and service, social support, money and material resources)	Personal goals Physiological status Functioning Health care use Perceptions of quality	This is an evolution of the Self-regulation model of patient management of asthma [38]
Self and family management framework revised [42]	The model is assumed to be recursive; outcomes influence further self and family management. Proximal outcomes can be seen as mediators of the outcomes of self- and family management.	Processes: Focusing on illness needs (learning, taking ownership, health promotion); Activating resources (health care, psychological, spiritual, social, community) Living with the condition (processing emotions, adjusting, integration with life, making meaning)	Conceptual model for patients living with chronic conditions and their families.	Facilitators and barriers: Personal and lifestyle factors (knowledge, beliefs, emotions, motivations, life patterns); Health status (co-morbidity, condition severity, symptoms/side effects, cognitive function); Resources (financial, equipment, community) Environment (home, work, community); Health care system (access, navigation, continuity of care, provider relationships).	Proximal outcomes: Behaviors (adherence, diet, physical activity, sleep); Cognitions (self-efficacy, motivation, perceived stress); Biomarkers (stress, inflammation, gene X environment); Symptom management (pain, fatigue). Distal outcomes: Health status (control, morbidity, mortality); Individual outcomes (quality of life, function); Family outcomes (quality of life, function); Health care (access, utilization, provider relationships, cost-effectiveness).	A synthesis of previous studies was used to update the original framework from the same author
The theoretical model of compliance in young diabetics [38]	Compliance is an active, intentional, and responsible Process. The control of diabetes explains compliance by way of the energy and will power for care.	Compliance: Self-care behavior Responsibility Intention Collaboration with physician	Conceptual model about compliance in adolescents living with diabetes	Factors directly affecting compliance: Motivation Energy and will-power Experience of results Sense of normality Fear Factors indirectly affecting compliance: Fear of complications Encouragement Support from parents Control of diabetes	Control of diabetes	
Adolescent Self-Regulation as a Foundation for Chronic Illness Self-Management [48]	Self-regulation is the ability to modulate cognition, emotion, and behavior to reach a goal. It is a foundation for individual and interpersonal processes in chronic illness self-management. Chronic Illness and related stress can influence further development of self-regulation or be influenced by the previous developmental history.	Family social environment Stress/regulatory Systems Self-regulation Chronic Illness and related stress Chronic illness self-management	Conceptual model about self-regulation in adolescents living with chronic conditions	Processes facilitating chronic illness self-management: Individual processes Interpersonal processes (family, community, healthcare system)	Individual and interpersonal goals Health	
Pediatric self-management model [27]	Self-management is the “interaction of health behaviors and related processes that patients and families engage in to care for chronic condition” Adherence is “the extent to which person’s behavior coincides with medical or health advice” “The degree to which self-management behaviors affect adherence, and ultimately outcomes, may result in changes in self-management behaviors.”	Self-management behaviors within the 4 domains: Individual Family Community Health-care system Adherence frequency: Treatments (medications, airway clearance, physical therapy, vitamin/mineral supplements, supplemental feeds) Lifestyle modifications (exercise, diet, fluid, sleep) Clinic appointment attendance Symptoms monitoring	Comprehensive model of self-management for pediatric patients	Non-modifiable and modifiable influences of each domain. Domain-specific influences impact self-management through cognitive, emotional, and social processes.	Individual: Symptoms and symptom control Complications Quality of life School/work days Drug resistance Mortality Health care utilization (e.g., emergency room visits, hospitalizations) System:Clinical decision-making Financial costs (e.g., Insurance rates, usage) Treatment efficacy Health care delivery	
Common sense model of parent–child shared regulation [45]	The model is supposed to be recursive, outcomes are evaluated and, if they are unsuccessful, the illness representation is modified. The health threat brings to an illness representation.	Coping procedures or action plans: the individual’s self-regulatory plan to face the health threat. It might include action or inaction. Appraisal: the individual’s evaluation of the perceived success or failure of the self-regulatory plan Health threat Illness Representation (including both parent illness representation and child illness representation)	Theory based on “Common sense model of self-regulation of health and illness” [47] for pediatric asthma			Authors analyze and reformulate the pre-existent theory “Common sense model of self-regulation of health and illness” [47]
Theory of parent–child relational illness management [50]	Parent responses influence child responses to illness. Parents and child responses and relationship influences all illness management and outcomes, which further involves parental reaction, and the cycle goes on.	Parent responses to illness: Appease Helplessness Control Blame Child responses to illness: Deny Minimize Withdraw Resent	Theory of Parent–Child Relational Illness Management for pediatric patients living with a chronic condition	Parents’ experiences related to: Childhood Adulthood Child’s illness	Illness management efforts and outcomes	

## Supplementary Materials

The following are available online at https://www.mdpi.com/article/10.3390/ijerph18073513/s1, File S1: Search Strategy.

## Abbreviations

CYA	Children and young adults
PRISMA	Preferred Reporting Items for Systematic Reviews and Meta-Analyses
FMSF	Family Management Style Framework
