# Supplementary material for: Self-Care in Pediatric Patients with Chronic Conditions: A Systematic Review of Theoretical Models
Source: Int J Environ Res Public Health. 2021 Mar 28;18(7):3513. doi: 10.3390/ijerph18073513 (PMC8037526; doi:10.3390/ijerph18073513)
Supplement: Supplementary file 1 [file ijerph-18-03513-s001.pdf]

Supplementary file - Search strategy

I. PubMed

Date	Filters	Search statement
09/07/2019	all fields	#1: “self care” OR “self-care” OR selfcare OR “self management” OR “self-management” OR complian* OR adheren*
	all fields	#2: Theor* OR model* OR conceptual OR framework*
	all fields	#3: “Chronic Disease” OR “Chronic Disease*” OR “Chronic* Ill*”
	title/abstract	#4: Pediatr* OR Paediatr* OR Newborn OR Newborn* OR Infant OR Infant* OR Child OR Child* OR Preschool OR adolescent OR adolescen* OR teenager* OR teen-ager* OR “young adult” OR “young adult*”
	/	#5: #1 AND #2 AND #3 AND #4

II. Scopus

Date	Filters	Search statement
09/07/2019	title/abstract/keywords	#1: “self care” OR “self-care” OR selfcare OR “self management” OR “self-management” OR complian* OR adheren*
	title/abstract/keywords	#2: Theor* OR model* OR conceptual OR framework*
	title/abstract/keywords	#3: “Chronic Disease” OR “Chronic Disease*” OR “Chronic* Ill*”
	title	#4: Pediatr* OR Paediatr* OR Newborn OR Newborn* OR Infant OR Infant* OR Child OR Child* OR Preschool OR adolescent OR adolescen* OR teenager* OR teen-ager* OR “young adult” OR “young adult*”
	/	#5: #1 AND #2 AND #3 AND #4

III. Cochrane

Date	Filters	Search statement
09/07/2019	/	#1: “self care” OR “self-care” OR selfcare OR “self management” OR “self-management” OR complian* OR adheren*
	/	#2: Theor* OR model* OR conceptual OR framework*
	/	#3: “Chronic Disease” OR “Chronic Disease*” OR “Chronic* Ill*”
	/	#4: Pediatr* OR Paediatr* OR Newborn OR Newborn* OR Infant OR Infant* OR Child OR Child* OR Preschool OR adolescent OR adolescen* OR teenager* OR teen-ager* OR “young adult” OR “young adult*”
	/	#5: #1 AND #2 AND #3 AND #4

IV. CINAHL

Date	Filters	Search statement
09/07/2019	/	#1: “self care” OR “self-care” OR selfcare OR “self management” OR “self-management” OR complian* OR adheren*
	/	#2: Theor* OR model* OR conceptual OR framework*
	/	#3: “Chronic Disease” OR “Chronic Disease*” OR “Chronic* Ill*”
	/	#4: Pediatr* OR Paediatr* OR Newborn OR Newborn* OR Infant OR Infant* OR Child OR Child* OR Preschool OR adolescent OR adolescen* OR teenager* OR teen-ager* OR “young adult” OR “young adult*”
	/	#5: #1 AND #2 AND #3 AND #4

V. EMBASE

Date	Filters	Search statement
09/07/2019	all fields	#1: “self care” OR “self-care” OR selfcare OR “self management” OR “self-management” OR complian* OR adheren*
	all fields	#2: Theor* OR model* OR conceptual OR framework*
	all fields	#3: “Chronic Disease” OR “Chronic Disease*” OR “Chronic* Ill*”
	title/abstract/author/key words	#4: Pediatr* OR Paediatr* OR Newborn OR Newborn* OR Infant OR Infant* OR Child OR Child* OR Preschool OR adolescent OR adolescen* OR teenager* OR teen-ager* OR “young adult” OR “young adult*”
	/	#5: #1 AND #2 AND #3 AND #4

VI. ISI – Web Of Science

Date	Filters	Search statement
09/07/2019	topic	#1: “self care” OR “self-care” OR selfcare OR “self management” OR “self-management” OR complian* OR adheren*
	topic	#2: Theor* OR model* OR conceptual OR framework*
	topic	#3: “Chronic Disease” OR “Chronic Disease*” OR “Chronic* Ill*”
	title	#4: Pediatr* OR Paediatr* OR Newborn OR Newborn* OR Infant OR Infant* OR Child OR Child* OR Preschool OR adolescent OR adolescen* OR teenager* OR teen-ager* OR “young adult” OR “young adult*”
	/	#5: #1 AND #2 AND #3 AND #4

VII. JBI - Joanna Briggs Institute

Date	Filters	Search statement
09/07/2019	all fields	#1: "self care" OR "self-care" OR selfcare OR "self management" OR "self-management" OR complian* OR adheren*
	all fields	#2: Pediatr* OR Paediatr* OR Newborn OR Newborn* OR Infant OR Infant* OR Child OR Child* OR Preschool OR adolescent OR adolescen* OR teenager* OR teen-ager* OR "young adult" OR "young adult*"
	/	#3: #1 AND #2

VIII. PsycINFO

Date	Filters	Search statement
09/07/2019	/	#1: "self care" OR "self-care" OR selfcare OR "self management" OR "self-management" OR complian* OR adheren*
	/	#2: Theor* OR model* OR conceptual OR framework*
	/	#3: "Chronic Disease" OR "Chronic Disease*" OR "Chronic* Ill*"
	/	#4: Pediatr* OR Paediatr* OR Newborn OR Newborn* OR Infant OR Infant* OR Child OR Child* OR Preschool OR adolescent OR adolescen* OR teenager* OR teen-ager* OR "young adult" OR "young adult*"
	/	#5: #1 AND #2 AND #3 AND #4

IX. PsycARTCLES

Date	Filters	Search statement
09/07/2019	/	#1: "self care" OR "self-care" OR selfcare OR "self management" OR "self-management" OR complian* OR adheren*
	/	#2: "Chronic Disease" OR "Chronic Disease*" OR "Chronic* Ill*"
	/	#3: Pediatr* OR Paediatr* OR Newborn OR Newborn* OR Infant OR Infant* OR Child OR Child* OR Preschool OR adolescent OR adolescen* OR teenager* OR teen-ager* OR "young adult" OR "young adult*"
	/	#4: #1 AND #2 AND #3
